# Thyroid function and outcomes in heart failure with mildly reduced ejection fraction: insights from a large, retrospective registry

**DOI:** 10.1007/s12020-026-04660-1

**Published:** 2026-07-13

**Authors:** Mohammad Abumayyaleh, Ibrahim Akin, Michelle Goertz, Marielen Reinhardt, Noah Abel, Alexander Schmitt, Felix Lau, Kathrin Weidner, Jonas Dudda, Henning Johann Steffen, Daniel Duerschmied, Michael Behnes, Tobias Schupp

**Affiliations:** Department of Cardiology, Haemostaseology and Medical Intensive Care, University Medical Centre Mannheim, Medical Faculty Mannheim, Heidelberg University, Mannheim, Germany

**Keywords:** HFmrEF, euthyroid, subclinical hypothyroidism, subclinical hyperthyroidism, low T3, MACCE, mortality.

## Abstract

**Objective:**

This study aimed to investigate the prognostic impact of thyroid function on the outcomes of patients with heart failure with mildly reduced ejection fraction (HFmrEF).

**Background:**

Thyroid function is crucial for the optimal performance of the cardiovascular system. However, the prognostic impact of subclinical thyroid dysfunction in patients with heart failure (HF), specifically HFmrEF, remains unclear.

**Methods:**

For the present study, patients hospitalized with HFmrEF and known thyroid function were included from 2016 to 2022. Patients were divided into four groups: euthyroid (reference group, n = 1186 [TSH 0.45–4.5 mU/L]), subclinical hypothyroidism (n = 95 [TSH > 4.5 mU/L, fT4 7.5-23 pmol/L]), subclinical hyperthyroidism (n = 139 [TSH < 0.45 mU/L, fT4 7.5-23 pmol/L]), and low T3 syndrome (n = 148 [fT3 ≤ 3.3 pmol/L]). Patients with manifest hyper- and hypothyroidism were excluded related to the low sample size. The primary endpoint was all-cause mortality at 30 months (median follow-up). Key secondary endpoint was HF-related rehospitalization at 30 months.

**Results:**

From 1568 patients with HFmrEF, most patients presented with euthyroidism (75.6%), followed by low T3 syndrome (9.4%), subclinical hyperthyroidism (8.9%), and subclinical hypothyroidism (6.1%). The risk of all-cause mortality at 30 months was highest in patients with low T3 syndrome (51.4%), followed by subclinical hypothyroidism (38.9%) and subclinical hyperthyroidism (36.7%), whereas euthyroid patients (26.6%) had the lowest risk of long-term all-cause mortality (p = 0.001). In multivariable Cox regression analyses, subclinical hypothyroidism (hazard ratio [HR] 1.454, 95% confidence interval [CI] 1.001–2.113, p = 0.049), subclinical hyperthyroidism (HR 1.458, 95% CI 1.046–2.033, p = 0.026), and low T3 syndrome (HR 1.594, 95% CI 1.185–2.145, p = 0.002) were identified as independent predictors of all-cause mortality at 30 months compared to euthyroid patients. Patients with low T3 syndrome were even associated with impaired prognosis as compared to patients with subclinical hyperthyroidism (HR 1.553, 95% CI 1.088–2.212; p = 0.015). However, thyroid status was not associated with the risk of HF-related rehospitalization at 30 months.

**Conclusion:**

In patients with HFmrEF, the presence of subclinical hypothyroidism, subclinical hyperthyroidism, and low T3 syndrome were identified as predictors of all-cause mortality, but not of HF-related rehospitalization at 30 months.

## Introduction

Heart failure (HF) is a major global health burden, affecting approximately 15 million individuals in Europe and an estimated 64.3 million people worldwide [1, 2]. It is characterized as a progressive, chronic degenerative condition that imposes substantial healthcare costs, particularly in the context of an aging population [3]. Among various physiological systems affecting the development and the progression of HF, thyroid hormone status has garnered increasing attention due to its potential impact on cardiovascular function. Thyroid function plays a pivotal role in modulating cardiovascular physiology, including cardiac contractility, electrophysiological activity, and influence lipid metabolism [4, 5]. In preclinical HF models, the administration of triiodothyronine (T3) was demonstrated to improve left ventricular ejection function (LVEF), myocardial remodeling, metabolic status, calcium handling, and overall cardiac performance. These studies also reported a reduction in the inducibility of atrial fibrillation (AF) [6–9]. Despite promising experimental findings, randomized clinical trials in HF patients have yielded inconsistent results regarding the benefits of thyroid hormone therapy, with some showing potential positive effects and others demonstrating no significant benefit [10–15]. Registry data have shown that both subclinical hypothyroidism and hyperthyroidism are associated with increased mortality risk in HF patients [16, 17]. Notably, the SCD-HeFT trial reported that abnormal thyroid function in patients with symptomatic HF with reduced ejection fraction (HFrEF) correlated with an increased mortality risk [18]. Similarly, in patients with HF with preserved ejection fraction (HFpEF), both hypothyroid and hyperthyroid states were linked to a higher risk of death compared to euthyroid individuals [19].

However, data on the prognostic implications of thyroid dysfunction in patients with HF with mildly reduced ejection fraction (HFmrEF) are limited. The present study aims to address this knowledge gap by analyzing a large HFmrEF population, with a particular focus on cardiovascular outcomes in relation to thyroid hormone status.

## Methods

### Study design

For this study, all consecutive patients hospitalized with HFmrEF at our medical center between January 2016 and December 2022 were included. This analysis is based on data from the HARMER registry (Heart Failure with Mildly Reduced Ejection Fraction Registry), a retrospective, single-center, all-comers cohort comprising consecutive patients hospitalized with HFmrEF at the University Medical Centre Mannheim, Germany (ClinicalTrials.gov Identifier: NCT05603390). Clinical and demographic data related to the index hospitalization were systematically collected using the hospitals’s electronic health records system. The registry was conducted in accordance with the principles outlined in the Declaration of Helsinki and was approved by the medical ethics committee II of the Medical Faculty Mannheim, University of Heidelberg (Ethics Approval Code: 2022–818).

### Inclusion and exclusion criteria

All patients aged 18 years or older hospitalized with HFmrEF were included. The diagnosis of HFmrEF was established retrospectively based on the 2021 ESC guidelines for the diagnosis and treatment of acute and chronic HF. In line with these criteria, patients with a LVEF between 41 and 49%, along with signs and/or symptoms of HF, were eligible for inclusion [20]. This cohort comprised patients presenting with de novo HFmrEF, as well as patients with a prior history of HFrEF or HFpEF who subsequently transitioned into the HFmrEF range. Patients with HF phenotypes outside the defined LVEF range were excluded from the study. For the present study, patients with incomplete assessment of thyroid function were excluded.

### Classification of thyroid function and echocardiographic assessment

**Fig. 1:**
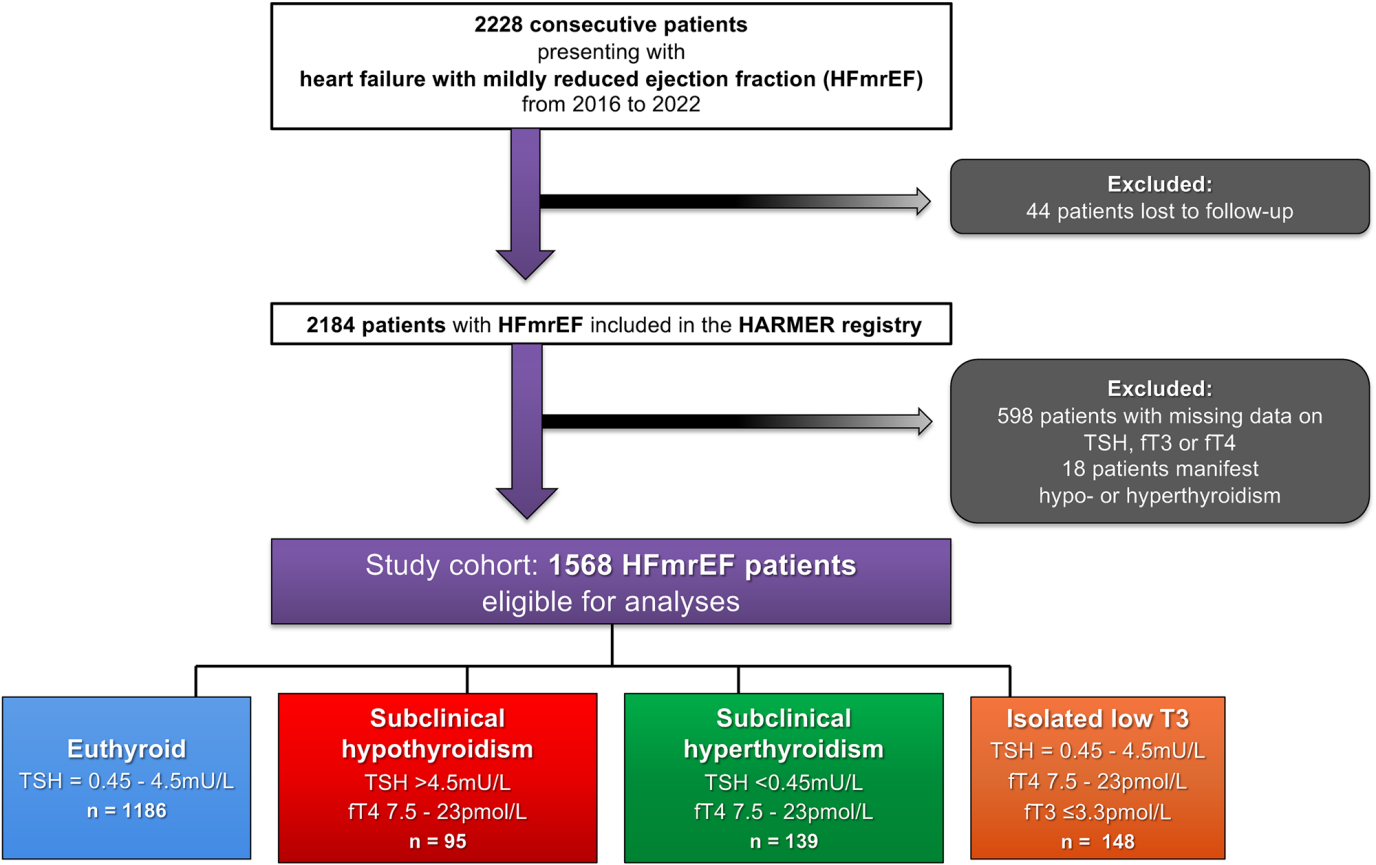
Study design

Thyroid function was assessed in all patients based on serum levels of thyroid-stimulating hormone (TSH) and free thyroxine (fT4). Low T3 syndrome based on free triiodothyronine (fT3). Classification into thyroid function groups was based on established laboratory reference ranges. Patients with TSH levels within the normal range (0.45–4.5 mU/L) and normal fT4 (7.5–23 pmol/L) concentrations were classified as euthyroid. Subclinical hypothyroidism was defined as an elevated TSH level (>4.5 mU/L) in the presence of normal fT4 value without overt clinical symptoms. Subclinical hyperthyroidism was diagnosed in patients with suppressed TSH levels (<0.45 mU/L) but normal fT4 concentration with the absence of typical clinical manifestations. Patients with reduced fT3 levels (≤3.3 pmol/L) in the setting of normal TSH and fT4 were categorized as having isolated low T3 syndrome. The classification was applied regardless of pre-existing thyroid disease or ongoing thyroid-related therapy. Patients with manifest hypo- and hyperthyroidism were excluded related to the low sample size (Fig. 1).

As part of standard clinical care, each patient underwent at least one transthoracic echocardiographic (TTE) examination performed by experienced cardiologists during the index hospitalization. All echocardiographic assessments were conducted in accordance with current European Society of Cardiology (ESC) guidelines, ensuring standardized evaluation of cardiac function [21]. LVEF is defined as the volume of blood ejected during left ventricular contraction and is measured using the Simpson biplane method. Tricuspid annular plane systolic excursion (TAPSE) performs right ventricular longitudinal function and is measured by M-mode echocardiography between end-diastole and peak systole with the cursor along the tricuspid lateral annulus in the apical four-chamber view.

### End points

The primary endpoint was long-term all-cause mortality at 30 months (median follow-up). Secondary endpoints included all-cause mortality occurring in-hospital and at 12 months. For in-hospital mortality, cardiac and non-cardiac causes were evaluated separately. Additional secondary endpoints comprised rehospitalizations due to HF at both 12 and 30 months. Further outcomes assessed at 30 months included cardiac-specific rehospitalization, coronary revascularization, acute myocardial infarction (AMI), stroke, and major adverse cardiac and cerebrovascular events (MACCE).

### Statistical analysis

Quantitative variables are reported as median (interquartile range [IQR]) according to their distribution. Non-normally distributed variables were compared using the Mann–Whitney U test. Categorical variables are expressed as absolute numbers and percentages, and compared using the chi-square or Fisher’s exact test, as appropriate. Survival analyses were performed using Kaplan–Meier estimates stratified by thyroid status, with univariable hazard ratios (HRs) and 95% confidence intervals (CIs) calculated. The prognostic impact of thyroid function was assessed using multivariable Cox proportional hazards regression, with results presented as forest plots. Pre-specified subgroup analyses were conducted with multivariable Cox regression, adjusting for age, male sex, body mass index (BMI), coronary artery disease (CAD), prior congestive HF, chronic kidney disease (CKD), diabetes mellitus, ischemic cardiomyopathy, AMI during index hospitalization, acute decompensated HF at index hospitalization, anemia (hemoglobin <12 g/dL in women and <13 g/dL in men), TAPSE, moderate-to-severe aortic stenosis, and mitral regurgitation. Multivariable Cox regression analyses were performed for each thyroid function category (subclinical hypothyroidism, subclinical hyperthyroidism, isolated low T3) to evaluate individual prognostic effects. A two-sided *P* value ≤ 0.05 was considered statistically significant. Statistical analyses were performed using SPSS, version 28 (IBM, Armonk, NY, USA).

## Results

### Patient characteristic and HF related data

Patients (n = 1568) were divided into four groups according to thyroid function: euthyroid (n = 1186; 75.6%), subclinical hypothyroidism (n = 95; 6.1%), subclinical hyperthyroidism (n = 139; 8.9%), and isolated low T3 syndrome (n = 148; 9.4%). Age, presented as median (IQR), was comparable across thyroid function groups: euthyroid 75 years (63–82), subclinical hypothyroidism 74 years (66–82), subclinical hyperthyroidism 77 years (69–83), and isolated low T3 77 years (66–83). Patients with subclinical hypo- and hyperthyroidism as well as low T3 syndrome were significantly less likely to be males compared to euthyroid patients (euthyroid: 66.3% vs. subclinical hypothyroidism: 48.4% vs. subclinical hyperthyroidism: 54.0% vs. isolated low T3: 57.4%; P = 0.001). These three groups also had a lower median BMI compared to euthyroid patients (27.0 kg/m² vs. 24.6 kg/m² vs. 26.1 kg/m² vs. 25.4 kg/m²; P = 0.001). Regarding comorbidities, CKD and malignancies were more prevalent in patients with subclinical hypothyroidism and low T3 (CKD: 27.1 vs. 41.1 vs. 26.6 vs. 39.9%; malignancy: 11.6 vs. 20.0 vs. 18.0 vs. 25.0%; P = 0.001 for both). Chronic obstructive pulmonary disease (COPD) was also significantly more common in these groups (9.8 vs. 13.7 vs. 11.5 vs. 18.9%; P = 0.007). During index hospitalization, acute decompensated HF was more frequently observed in patients with subclinical hypothyroidism and low T3 syndrome compared to euthyroid and subclinical hyperthyroidism patients (19.2 vs. 33.7 vs. 16.5 vs. 33.1%; P = 0.001). Other characteristics such as clinical findings, medical history, cardiovascular risk factors, comorbidities, and medications during index hospitalization are presented in Table 1.

**Table 1:** Baseline characteristics

	Euthyroid n = 1186	Subclinical hypothyroidism n = 95	Subclinical hyperthyroidism n = 139	Low T3 n = 148	p value
**Age**, median (IQR)	75 (63-82)	74 (66-82)	77 (69-83)	77 (66-83)	0.101
**Male sex**, n (%)	786 (66.3)	46 (48.4)	75 (54.0)	85 (57.4)	**0.001**
**Body mass index**, kg/m^2^, median (IQR)	27.0 (24.2-31.0)	24.6 (22.5-28.4)	26.1 (23.5-29.0)	25.4 (22.3-30.5)	**0.001**
**SBP**, mmHg, median (IQR)	145 (130-166)	139 (117-169)	146 (125-166)	125 (110-146)	**0.001**
**DBP**, mmHg, median (IQR)	80 (70-92)	74 (66-88)	80 (70-88)	70 (60-83)	**0.001**
**Heart rate**, bpm, median (IQR)	80 (69-94)	81 (68-97)	80 (69-92)	84 (70-105)	0.311
**Medical history**, n (%)					
Coronary artery disease	452 (38.1)	36 (37.9)	56 (40.3)	63 (42.6)	
Prior myocardial infarction	268 (22.6)	24 (25.3)	33 (23.7)	37 (25.0)	0.861
Prior PCI	323 (27.2)	24 (25.3)	36 (25.9)	39 (26.4)	0.962
Prior CABG	113 (9.5)	6 (6.3)	15 (10.8)	18 (12.2)	0.476
Prior valvular surgery	59 (5.0)	3 (3.2)	5 (3.6)	5 (3.4)	0.647
Congestive heart failure	372 (31.4)	38 (40.0)	45 (32.4)	57 (38.5)	0.136
Prior decompensated heart failure (<12 months)	115 (9.7)	16 (16.8)	12 (8.6)	13 (8.8)	0.132
Prior ICD	26 (2.2)	2 (2.1)	2 (1.4)	1 (0.7)	0.617
Prior sICD	6 (0.5)	1 (1.1)	0 (0.0)	0 (0.0)	0.538
Prior CRT-D	15 (1.3)	1 (1.1)	1 (0.7)	1 (0.7)	0.881
Prior Pacemaker	100 (8.4)	8 (8.4)	14 (10.1)	13 (8.8)	0.933
Chronic kidney disease	321 (27.1)	39 (41.1)	37 (26.6)	59 (39.9)	**0.001**
Peripheral artery disease	98 (8.3)	13 (13.7)	24 (17.3)	21 (14.2)	**0.001**
Stroke	186 (15.7)	19 (20.0)	22 (15.8)	24 (16.2)	0.746
Liver cirrhosis	17 (1.4)	2 (2.1)	3 (2.2)	6 (4.1)	0.148
Malignancy	138 (11.6)	19 (20.0)	25 (18.0)	37 (25.0)	**0.001**
COPD	116 (9.8)	13 (13.7)	16 (11.5)	28 (18.9)	**0.007**
**Cardiovascular risk factors**, n (%)					
Arterial hypertension	930 (78.4)	71 (74.7)	108 (77.7)	112 (75.7)	0.762
Diabetes mellitus	431 (36.3)	29 (30.5)	48 (34.5)	60 (40.5)	0.439
Hyperlipidemia	361 (30.4)	19 (20.0)	41 (29.5)	49 (33.1)	0.149
Smoking	434 (36.6)	36 (37.9)	44 (31.7)	53 (35.8)	0.694
Current	227 (19.1)	15 (15.8)	24 (17.3)	23 (15.5)	0.618
Former	207 (17.5)	21 (22.1)	20 (14.4)	30 (20.3)	0.387
Family history	123 (10.4)	6 (6.3)	7 (5.0)	10 (6.8)	0.081
**Comorbidities at index hospitalization**, n (%)					
Acute coronary syndrome					
Unstable angina	74 (6.2)	1 (1.1)	4 (2.9)	4 (2.7)	**0.028**
STEMI	80 (6.7)	11 (11.6)	7 (5.0)	13 (8.8)	0.198
NSTEMI	149 (12.6)	10 (10.5)	14 (10.1)	19 (12.8)	0.793
Acute decompensated heart failure at index	228 (19.2)	32 (33.7)	23 (16.5)	49 (33.1)	**0.001**
Cardiogenic shock	26 (2.2)	4 (4.2)	3 (2.2)	5 (3.4)	0.539
Atrial fibrillation	488 (41.1)	47 (49.5)	60 (43.2)	66 (44.6)	0.392
Cardiopulmonary resuscitation	27 (2.3)	4 (4.2)	2 (1.4)	6 (4.1)	0.320
Out-of-hospital	10 (0.8)	3 (3.2)	1 (0.7)	2 (1.4)	0.175
In-hospital	17 (1.4)	1 (1.1)	1 (0.7)	4 (2.7)	0.527
Stroke	237 (20.0)	7 (7.4)	42 (30.2)	4 (2.7)	**0.001**
**Medication on admission**, n (%)					
ACE-inhibitor	421 (35.5)	29 (30.5)	52 (37.4)	50 (33.8)	0.709
ARB	268 (22.6)	20 (21.1)	27 (19.4)	32 (21.6)	0.843
Beta-blocker	651 (54.9)	55 (57.9)	72 (51.8)	88 (59.5)	0.560
Aldosterone antagonist	99 (8.3)	8 (8.4)	25 (18.0)	8 (5.4)	**0.001**
ARNI	8 (0.7)	3 (3.2)	2 (1.4)	1 (0.7)	0.082
SGLT2-inhibitor	30 (2.5)	0 (0.0)	2 (1.4)	4 (2.7)	0.379
Loop diuretics	389 (32.8)	43 (45.3)	57 (41.0)	61 (41.2)	**0.009**
Statin	537 (45.3)	43 (45.3)	59 (42.4)	65 (43.9)	0.926
ASA	389 (32.8)	28 (29.5)	44 (31.7)	48 (32.4)	0.921
P2Y12-inhibitor	105 (8.9)	10 (10.5)	13 (9.4)	13 (8.8)	0.954
DOAC	296 (25.0)	24 (25.3)	29 (20.9)	24 (16.2)	0.096
Vitamin K antagonist	97 (8.2)	7 (7.4)	10 (7.2)	17 (11.5)	0.511

*ACE* angiotensin-converting-enzyme, *ARB* angiotensin receptor blocker, *ARNI* angiotensin receptor neprilysin inhibitor, *ASA* acetylsalicylic acid, *CABG* coronary artery bypass grafting, *CKD* chronic kidney disease, *COPD* chronic obstructive pulmonary disease, *CRT-D* cardiac resynchronization therapy with defibrillator, *DBP* diastolic blood pressure, *DOAC* directly acting oral anticoagulant, *IQR* interquartile range, *PCI* percutaneous coronary intervention, *(N)STEMI* non-ST-segment elevation myocardial infarction, *SBP* systolic blood pressure, *SGLT2* sodium glucose linked transporter 2, *(s) ICD* (subcutaneous) implantable cardioverter defibrillator.

Level of significance p ≤ 0.05. Bold type indicates statistical significance.

Consistent with the incidence of acute decompensated HF at index hospitalization, the rate of moderate-to-severe tricuspid regurgitation was significantly higher in patients with subclinical hypothyroidism and isolated low T3 compared to the other groups (14.7 vs. 21.1 vs. 9.4 vs. 22.3%; P = 0.007). Furthermore, levels of the amino-terminal prohormone of brain natriuretic peptide (NT-proBNP) were higher in these patients (2186 pg/mL [688–4872] vs. 4776 pg/mL [1601–12977] vs. 3157 pg/mL [1195–8281] vs. 4285 pg/mL [1965–10101]; P = 0.001). Levothyroxine use was more frequent in patients with subclinical hypothyroidism compared with euthyroid, subclinical hyperthyroidism, and low T3 syndrome (12.3 vs. 41.1 vs. 15.8 vs. 19.6%; P = 0.001). The distribution of HF etiology did not differ significantly among groups, with ischemic cardiomyopathy being the predominant cause. Renal function, assessed by eGFR, was significantly lower in patients with subclinical hypothyroidism and low T3 syndrome compared with euthyroid patients (68 [50–88] vs. 59 [44–81] vs. 64 [44–83] vs. 58 (35–87) mL/min/1.73 m²; P = 0.001).Hemoglobin levels showed a similar pattern, with reduced values in subclinical hypothyroidism and low T3 compared with the euthyroid group (13.1 [11.3–14.4] vs. 11.4 [9.3–13.5] vs. 12.3 [10.1–13.6] vs. 10.6 [9.2–12.1] g/dL; P = 0.001). Other HF-related and procedural data including medication at discharge are shown in Table 2.

**Table 2:** Heart failure-related and procedural data

	Euthyroid n = 1186	Subclinical hypothyroidism n = 95	Subclinical hyperthyroidism n = 139	Low T3 *n* = *148*	p value
**Heart failure etiology**, n (%)					
Ischemic cardiomyopathy	659 (55.6)	52 (54.7)	78 (56.1)	79 (53.4)	0.960
Primary non-ischemic cardiomyopathy	80 (6.7)	10 (10.5)	6 (4.3)	13 (8.8)	0.239
Hypertensive cardiomyopathy	109 (9.2)	6 (6.3)	13 (9.4)	8 (5.4)	0.370
Congenital heart disease	2 (0.2)	1 (1.1)	0 (0.0)	0 (0.0)	0.233
Valvular heart disease	55 (4.6)	5 (5.3)	4 (2.9)	5 (3.4)	0.692
Tachycardia associated	76 (6.4)	5 (5.3)	12 (8.6)	7 (4.7)	0.561
Tachymyopathy	19 (1.6)	0 (0.0)	6 (4.3)	2 (1.4)	0.060
Pacemaker-induced cardiomyopathy	10 (0.8)	1 (1.1)	1 (0.7)	3 (2.0)	0.564
Unknown	195 (16.4)	15 (15.8)	25 (18.0)	33 (22.3)	0.337
**NYHA functional class**, n (%)					
I/II	898 (75.8)	57 (60.0)	107 (77.0)	90 (60.8)	**0.001**
III	200 (16.9)	30 (31.6)	18 (12.9)	32 (21.6)	
IV	88 (7.4)	8 (8.4)	14 (10.1)	26 (17.6)	
**Echocardiographic data**					
LVEF, %, median (IQR)	45 (45–47)	45 (45–48)	45 (44–45)	45 (44–45)	**0.005**
IVSd, median (IQR)	12 (11–13)	11 (10–13)	12 (11–13)	11 (10–13)	0.139
LVEDD, mm, median (IQR)	49 (45–54)	48 (42–52)	49 (44–54)	48 (42–54)	0.239
TAPSE, mm, median (IQR)	20 (17–23)	20 (16–23)	20 (17–23)	19 (17–23)	0.704
LA diameter, mm, median (IQR)	41 (37–47)	42 (37–48)	44 (39–49)	43 (35–49)	0.168
LA surface, cm^2^, median (IQR)	21 (17–25)	20 (17–25)	21 (17–25)	24 (19–29)	0.124
Moderate-severe aortic stenosis, n (%)	127 (10.7)	8 (8.4)	11 (7.9)	16 (10.8)	0.689
Moderate-severe aortic regurgitation, n (%)	47 (4.0)	7 (7.4)	5 (3.6)	4 (2.7)	0.320
Moderate-severe mitral regurgitation, n (%)	140 (11.8)	14 (14.7)	12 (8.6)	23 (15.5)	0.267
Moderate-severe tricuspid regurgitation, n (%)	174 (14.7)	20 (21.1)	13 (9.4)	33 (22.3)	**0.007**
VCI, mm, median (IQR)	19 (15–24)	22 (14–27)	19 (13–21)	22 (16–29)	0.231
Aortic root, mm, median (IQR)	33 (30–36)	32 (28–36)	32 (29–35)	32 (29–35)	**0.008**
**Coronary angiography**, n (%)	507 (42.7)	38 (40.0)	43 (30.9)	59 (39.9)	0.061
No evidence of coronary artery disease	109 (21.5)	8 (21.1)	7 (16.3)	15 (25.4)	0.762
1-vessel disease	91 (17.9)	10 (26.3)	9 (20.9)	6 (10.2)	
2-vessel disease	104 (20.5)	7 (18.4)	11 (25.6)	13 (22.0)	
3-vessel disease	203 (40.0)	13 (34.2)	16 (37.2)	25 (42.4)	
Prior CABG	38 (7.5)	0 (0.0)	3 (7.0)	6 (10.2)	0.288
Chronic total occlusion	69 (13.6)	2 (5.3)	6 (14.0)	7 (11.9)	0.516
PCI, n (%)	261 (51.5)	20 (52.6)	20 (46.5)	29 (49.2)	0.916
Sent to CABG, n (%)	32 (6.3)	2 (5.3)	1 (2.3)	0 (0.0)	0.173
**Baseline laboratory values**, median (IQR)					
TSH, mU/L	1.4 (0.9–2.0)	7.1 (5.2–9.9)	0.3 (0.1–0.3)	1.5 (1.0–2.5)	**0.001**
fT4, pmol/L	15.0 (13.0–16.9)	14.3 (12.6–17.1)	16.9 (14.7–19.0)	14.7 (12.8–16.8)	**0.001**
fT3, pmol/L	4.1 (3.7–4.6)	3.1 (2.3–3.8)	3.5 (2.7–4.4)	2.6 (2.1–3.0)	**0.001**
Potassium, mmol/L	3.9 (3.6–4.2)	3.8 (3.4–4.2)	3.9 (3.7–4.2)	3.9 (3.5–4.2)	0.502
Sodium, mmol/L	139 (137–141)	139 (137–141)	140 (138–142)	139 (136–141)	0.276
Creatinine, mg/dL	1.0 (0.9–1.4)	1.1 (0.9–1.5)	1.1 (0.9–1.4)	1.1 (0.9–1.8)	0.082
eGFR, mL/min/1.73 m^2^	68 (50–88)	59 (44–81)	64 (44–83)	58 (35–87)	**0.001**
Hemoglobin, g/dL	13.1 (11.3–14.4)	11.4 (9-3-13.5)	12.3 (10.1–13.6)	10.6 (9.2–12.1)	**0.001**
WBC, x 10^9^/L	8.2 (6.5–9.8)	8.2 (6.3–10.3)	8.6 (6.7–11.0)	8.6 (6.0–11.4)	0.246
Platelet count, x 10^9^/L	224 (181–273)	245 (195–302)	220 (175–287)	233 (165–320)	0.189
HbA1c, %	5.9 (5.5–6.7)	5.8 (5.4–6.4)	5.8 (5.5–6.8)	6.0 (5.6–7.8)	0.466
LDL-cholesterol, mg/dL	100 (76–126)	97 (63-123)	98 (71–134)	82 (61–115)	**0.043**
HDL-cholesterol, mg/dL	42 (35–52)	43 (37–52)	42 (34–49)	46 (33–58)	0.459
C-reactive protein, mg/L	8.0 (2.9–31.0)	19.2 (7.9–42.2)	18.1 (5.8–55.2)	29.3 (11.0–73.9)	**0.001**
NT-proBNP, pg/mL	2186 (688–4872)	4776 (1601–12977)	3157 (1195–8281)	4285 (1965–10101)	**0.001**
NT-proBNP (eGFR corrected), pg/mL	1234 (522–2849)	2594 (1394–6176)	2179 (1129–4128)	2238 (1164–5786)	**0.001**
Cardiac troponin I, µg/L	0.02 (0.02–0.12)	0.05 (0.02–0.33)	0.03 (0.02–0.16)	0.06 (0.02–0.44)	**0.001**
**Medication at discharge**, n (%)					
Levothyroxine	146 (12.3)	39 (41.1)	22 (15.8)	29 (19.6)	**0.001**
ACE-inhibitor	586 (50.6)	45 (51.7)	68 (50.7)	65 (46.8)	0.847
ARB	279 (24.1)	17 (19.5)	24 (17.9)	29 (20.9)	0.300
Beta-blocker	893 (77.0)	67 (77.0)	101 (75.4)	100 (71.9)	0.593
Aldosterone antagonist	151 (13.0)	11 (12.6)	22 (16.4)	17 (12.2)	0.712
ARNI	12 (1.0)	3 (3.4)	3 (2.2)	1 (0.7)	0.157
SGLT2-inhibitor	54 (4.7)	1 (1.1)	5 (3.7)	5 (3.6)	0.433
Loop diuretics	486 (41.9)	50 (57.5)	64 (47.8)	86 (61.9)	**0.001**
Statin	836 (72.1)	49 (56.3)	101 (75.4)	78 (56.1)	**0.001**
Digitalis	58 (5.0)	1 (1.1)	11 (8.2)	6 (4.3)	0.124
Amiodarone	28 (2.4)	4 (4.6)	1 (0.7)	7 (5.0)	0.089
ASA	599 (51.7)	38 (43.7)	60 (44.8)	61 (43.9)	0.101
P2Y12-inhibitor	360 (31.1)	31 (35.6)	38 (28.4)	41 (29.5)	0.692
DOAC	387 (33.4)	35 (40.2)	51 (38.1)	38 (27.3)	0.147
Vitamin K antagonist	87 (7.5)	3 (3.4)	3 (2.2)	9 (6.5)	0.074

*ACE* angiotensin-converting enzyme, *ADHF* acute decompensated heart failure, *ARB* angiotensin receptor blocker, *ARNI* angiotensin receptor neprilysin inhibitor, *ASA* acetylsalicylic acid, *CABG* coronary artery bypass grafting, *DOAC* directly acting oral anticoagulant, *eGFR* estimated glomerular filtration rate, *fT3* free triiodothyronine, *fT4* free Thyroxine, *HbA1c* glycated hemoglobin, *HDL* high-density lipoprotein, *IQR* interquartile range, *IVSd* interventricular septum in diastole, *LA* left atrial, *LDL* low-density lipoprotein, *LVEDD* Left ventricular end-diastolic diameter, *LVEF* left ventricular ejection fraction, *NT-proBNP* amino-terminal prohormone of brain natriuretic peptide, *NYHA* New York Heart Association, *PCI* percutaneous coronary intervention, *TAPSE* tricuspid annular plane systolic excursion, *TSH* thyroid-stimulating hormone, *VCI* vena cava inferior, *WBC* white blood cell count.

Level of significance p ≤ 0.05. Bold type indicates statistical significance.

### Clinical outcomes

At 30 months, the risk of all-cause mortality was significantly higher in patients with altered thyroid function, particularly in the low T3 group, compared with euthyroid patients (26.6 vs. 38.9 vs. 36.7 vs. 51.4%; P = 0.001). In-hospital mortality rates, regardless of cause, were more frequent in subclinical hypothyroidism and isolated low T3 groups, specifically attributed to higher rates of cardiac death (Table 3). Similarly, the 12-month mortality rate was also significantly higher in these groups compared with euthyroid patients (16.4 vs. 33.7 vs. 29.5 vs. 40.5%; P = 0.001). The incidence of MACCE at 30 months was significantly higher in the subclinical hypothyroidism and isolated low T3 groups compared with euthyroid patients (34.3 vs. 43.2 vs. 41.0 vs. 56.1%; P = 0.001). Other secondary endpoints and follow-up data are presented in Table 3.

**Table 3:** Follow-up data, primary and secondary endpoints stratified by thyroid status

	Euthyroid n = 1186	Subclinical hypothyroidism n = 95	Subclinical hyperthyroidism n = 139	Low T3 n = 148	p value
**Primary endpoint**, n (%)					
All-cause mortality, at 30 months	315 (26.6)	37 (38.9)	51 (36.7)	76 (51.4)	**0.001**
**Secondary endpoints**, n (%)					
All-cause mortality, in-hospital	27 (2.3)	8 (8.4)	5 (3.6)	9 (6.1)	**0.001**
Cardiac mortality, in-hospital	8 (0.7)	3 (3.2)	1 (0.7)	5 (3.4)	**0.004**
Non-cardiac mortality, in-hospital	19 (1.6)	5 (5.3)	4 (2.9)	4 (2.7)	0.076
All-cause mortality, at 12 months	194 (16.4)	32 (33.7)	41 (29.5)	60 (40.5)	**0.001**
Heart failure-related rehospitalization, at 12 months	100 (8.6)	12 (13.8)	12 (9.0)	19 (13.7)	0.123
Heart failure-related rehospitalization, at 30 months	141 (12.2)	14 (16.1)	13 (9.7)	24 (17.3)	0.178
Cardiac rehospitalization, at 30 months	247 (21.3)	20 (23.0)	23 (17.2)	32 (23.0)	0.624
Coronary revascularization, at 30 months	81 (7.0)	5 (5.7)	7 (5.2)	5 (3.6)	0.417
Acute myocardial infarction, at 30 months	34 (2.9)	2 (2.3)	2 (1.5)	3 (2.2)	0.755
Stroke, at 30 months	37 (3.2)	1 (1.1)	1 (0.7)	4 (2.9)	0.313
MACCE, at 30 months	407 (34.3)	41 (43.2)	57 (41.0)	83 (56.1)	**0.001**
**Follow-up data**, median (IQR)					
Hospitalization time, days	8 (5–12)	10 (5–22)	11 (7–18)	13 (9–21)	**0.001**
ICU time, days	0 (0–0)	0 (0–2)	0 (0–0)	0 (0–1)	**0.001**
Follow-up time, days	928 (458–1682)	699 (256–1537)	850 (250–1614)	535 (134–1262)	**0.001**

*ADHF* acute decompensated heart failure, *CI* confidence interval, *HF* heart failure, *HR* hazard ratio, *ICU* intensive care unit, *IQR* interquartile range, *MACCE* major adverse cardiac and cerebrovascular events.

Level of significance p ≤ 0.05. Bold type indicates statistical significance.

**Fig. 2:**
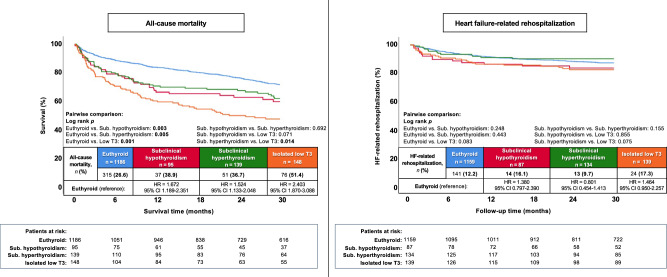
Kaplan-Meier curves for all-cause mortality and heart failure-related rehospitalization

**Fig. 3:**
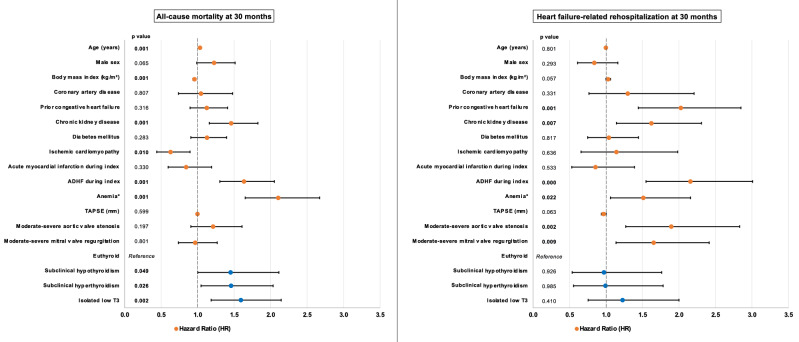
Predictors for all-cause mortality and heart failure-related rehospitalization at 30 months

In multivariable Cox regression analyses, subclinical hypothyroidism (HR 1.454, 95% CI 1.001–2.113, P = 0.049), subclinical hyperthyroidism (HR 1.458, 95% CI 1.046–2.033, P = 0.026), and isolated low T3 (HR 1.594, 95% CI 1.185–2.145, P = 0.002) were identified as independent predictors of all-cause mortality at 30 months compared with euthyroid status. Furthermore, isolated low T3 was also associated with worse prognosis compared with subclinical hyperthyroidism (HR 1.553, 95% CI 1.088–2.212; P = 0.015). However, thyroid status was not associated with the risk of HF-related rehospitalization at 30 months. Kaplan–Meier survival curves for all-cause mortality and HF–related rehospitalization are shown in Fig. 2. The predictors of all-cause mortality and HF–related rehospitalization at 30 months are summarized in Fig. 3.

## Discussion

The main findings of the present study, investigating the prognostic impact of thyroid function on the outcomes of patients with HFmrEF can be summarized as follows: (1) The risk of 30-month all-cause mortality was highest in HFmrEF patients with isolated low T3; (2) Subclinical hypothyroidism, subclinical hyperthyroidism, and isolated low T3 were identified as independent predictors of all-cause mortality at 30 months; (3) Isolated low T3 was associated with the highest risk of long-term all-cause mortality in HFmrEF; (4) Thyroid status did not impact the risk of HF-related rehospitalization at 30 months.

Thyroid function is essential for cellular homeostasis and overall metabolism [22]. Furthermore, thyroid hormones play a key role in regulating the autonomic nervous system and the renin–angiotensin–aldosterone system (RAAS) [23]. They also influence the gene expression of β1-adrenergic receptors, thereby enhancing heart rate and myocardial contractility [24]. In addition, thyroid hormones affect vascular smooth muscle cells, vascular tone, and the metabolic profile. Through these mechanisms, they exert significant effects on coronary artery function and contribute to the development and progression of HF [23]. Furthermore, thyroid function disorders are common, affecting nearly 11% of the adult population in Europe, with 85.2% of cases representing subclinical dysfunction [25]. In our analysis, such disorders were observed in 24.4% of patients with HFmrEF, despite the exclusion of manifest thyroid disorders due to their small sample size.

In our analysis, clinical outcomes, including in-hospital, long-term mortality, and MACCE, were more frequent in HFmrEF patients with subclinical hypothyroidism as well as those with isolated low T3 syndrome. Independent of amiodarone therapy, a high prevalence of both conditions has previously been reported in patients with HF [26]. For isolated low T3 syndrome, previous studies have identified it as an independent predictor of all-cause mortality in hospitalized patients with HF, a finding that we confirm in our HFmrEF cohort [27, 28]. Furthermore, isolated low T3 was associated with the highest risk of long-term all-cause mortality in our patients, which may be related to myocardial fibrosis and impaired myocardial perfusion linked to low T3 levels [29]. For subclinical hypothyroidism, studies in patients with HFrEF have demonstrated an association with poor prognosis, and in older individuals, an increased risk of incident HF has been observed [30, 31]. Subclinical hypothyroidism contributes to endothelial dysfunction and appears to be associated with dyslipidemia and hypertension [32, 33]. Furthermore, an association between manifest hypothyroidism and atherosclerosis has also been described, with the subclinical form considered a potential precursor stage [34]. Furthermore, thyroid dysfunction, such as isolated low T3 and subclinical hypothyroidism, was highly prevalent in patients with CKD and active malignancy in our cohort. The pathophysiology of thyroid dysfunction in these diseases is likely multifactorial. The causal relationship between these conditions and thyroid dysfunction may be bidirectional [35]. In particular, isolated low T3 may represent a consequence of advanced systemic disease in the context of non-thyroidal illness syndrome (NTIS) [36, 37]. However, data on clinical outcomes in HFmrEF patients are limited.

In addition, the present study identified subclinical hyperthyroidism as an independent predictor of mortality. Patients with this condition have been reported to carry an increased risk of CAD and HF [23, 38]. HF remains the most common complication in hyperthyroidism, with up to 50% of affected patients demonstrating reduced LVEF [18]. In HFrEF, hyperthyroidism has been associated with an 85% relative increase in mortality, whereas in our HFmrEF cohort, a 52% higher relative risk of mortality was observed compared with euthyroid patients [39]. Hyperthyroidism increases cardiac output through its effects on heart rate and stroke volume, enhances myocardial contractility, and alters myosin heavy chain isoform expression from the β- to the α-form [40]. Excess thyroid hormones also increase blood volume and venous return via neurohormonal activation, while reducing systemic vascular resistance, leading to circulatory congestion and potential decompensation, particularly in patients with de novo HF [41]. In this setting, activation of RAAS promotes further fluid retention, which, if untreated, may result in systolic dysfunction. Additionally, hyperthyroidism is a well-recognized trigger for cardiac arrhythmias, further contributing to adverse outcomes [42]. HF-related hospitalizations have been reported to occur more frequently in patients with both subclinical hypothyroidism and hyperthyroidism [31]; however, this observation could not be confirmed in our analysis.

This subanalysis has several limitations. First, due to its retrospective and single-center design, the results may be influenced by both measured and unmeasured confounding, including factors such as disease severity and concomitant medications affecting thyroid function. Second, data on the impact of thyroid hormone therapy on outcomes were not available, nor were follow-up TSH measurements. Third, we did not have information regarding the underlying causes of thyroid dysfunction. NT-proBNP measurements were not systematically available in all patients. Furthermore, data on the causes of death could not be obtained for all patients. Fourth, it cannot be excluded that normal-range T4 levels were the result of levothyroxine supplementation in euthyroid group. Fifth, thyroid status classification was based solely on biochemical parameters (TSH, fT3, and fT4), irrespective of pre-existing thyroid disease or ongoing thyroid-related therapy. In this regard, 12.3% of patients classified as euthyroid were discharged on levothyroxine, suggesting that thyroid dysfunction was actively managed in a subset of the cohort. Levothyroxine supplementation may have influenced the measured biochemical thyroid parameters and potentially led to misclassification, as treatment could normalize hormone levels in patients who would otherwise be classified as hypothyroid. This may have biased the observed associations, most likely toward the null, by attenuating differences between groups. Sixth, data on recent exposure to iodinated contrast agents were not available, which may have influenced thyroid function assessment in a subset of patients. Nevertheless, to the best of our knowledge, this is one of the largest monocentric studies to investigate thyroid function in patients with HFmrEF.

In conclusion, in-hospital, 12-month, and 30-month mortality rates were higher in HFmrEF patients with subclinical hypothyroidism and isolated low T3. All forms of subclinical thyroid dysfunction were independent predictors of all-cause mortality at 30 months, with isolated low T3 associated with the poorest prognosis. Further studies are warranted to determine optimal treatment strategies for these conditions in patients with HFmrEF in order to improve clinical outcomes.

## Data Availability

The datasets generated and analyzed during the current study are available from the corresponding author upon reasonable request.
